# Comparing the application of mNGS after combined pneumonia in hematologic patients receiving hematopoietic stem cell transplantation and chemotherapy: A retrospective analysis

**DOI:** 10.3389/fcimb.2022.969126

**Published:** 2022-09-21

**Authors:** Binglei Zhang, Ruirui Gui, Qian Wang, Xueli Jiao, Zhen Li, Juan Wang, Lu Han, Ling Zhou, Huili Wang, Xianjing Wang, Xinxin Fan, Xiaodong Lyu, Yongping Song, Jian Zhou

**Affiliations:** ^1^ Department of Hematology, Affiliated Cancer Hospital of Zhengzhou University and Henan Cancer Hospital, Zhengzhou, China; ^2^ School of Basic Medical Sciences, Academy of Medical Sciences, Henan Academy of Medical and Pharmaceutical Sciences, Zhengzhou University, Zhengzhou, China; ^3^ Department of Hematology, The Third People’s Hospital of Zhengzhou, Zhengzhou, China

**Keywords:** metagenomic next generation sequencing (mNGS), hematopoietic stem cell transplantation, chemotherapy, bronchoalveolar lavage fluid (BALF), pathogen

## Abstract

Rapid and accurate pathogen identification is essential for timely and effective treatment of pneumonia. Here, we describe the use of metagenomic next-generation sequencing (mNGS) of bronchoalveolar lavage (BALF) fluid to identify pathogens in patients with hematologic comorbid respiratory symptoms in a retrospective study with 84 patients. In the transplantation group, 8 cases (19.5%) and 47 cases (97.9%) were positive for BALF by conventional method detection and mNGS detection, respectively, and 6 cases (14.0%) and 41 cases (91.1%) in chemotherapy group, respectively. The detection rate of mNGS in both groups was significantly higher than that of conventional detection methods (all P<0.05). *Pseudomonas aeruginosa* and *Streptococcus pneumoniae* were the most common bacterial infections in the transplantation and chemotherapy groups, respectively. *Aspergillus* was the most common fungal infection in both groups. *Human betaherpesvirus 5 (HHV-5), torque teno virus and human betaherpesvirus 7 (HHV-7)* were the most common pathogen species in both groups. The most common type of infection in patients in the transplantation and chemotherapy groups was the mixed infection of bacteria-virus. Most patients in the transplantation group had mixed infections based on multiple viruses, with 42 cases of viral infections in the transplantation group and 30 cases of viral infections in the chemotherapy group, which were significantly higher in the transplantation group than in the chemotherapy group (χ2 = 5.766, P=0.016). and the mixed infection of virus-virus in the transplantation group was significantly higher than that in the chemotherapy group (27.1% *vs* 4.4%, P=0.003). The proportion of death due to pulmonary infection was significantly higher in the transplantation group than in the chemotherapy group (76.9% *vs* 16.7%, χ2 = 9.077, P=0.003). This study demonstrated the value of mNGS of BALF in improving the diagnosis and prognosis of hematologic comorbid pneumonia, helping patients to obtain timely and effective treatment, and giving guidance on the overall treatment plan for patients, with particular benefit for patients with hematologic chemotherapy comorbid pneumonia.

## 1 Introduction

Respiratory tract infection has long been the most common comorbidity in hematologic disease, especially in patients in the granulocyte-deficient phase of chemotherapy and those receiving allogeneic hematopoietic stem cell transplantation (allo-HSCT) (Garcia et al., 2013; Kim et al., 2013; Beam et al., 2018). Also, lower respiratory tract infection has become the leading cause of death in allo-HSCT (Nichols et al., 2004; Langelier et al., 2018; Zhou and Moore, 2021). mNGS requires no preconceptions, can detect multiple pathogens at once, and has been shown in several clinical studies to rapidly and accurately assist in the diagnosis of complex pulmonary infections and severe infections. In addition, mNGS is valuable in aiding the diagnosis of invasive fungal infections, mixed pulmonary infections and severe unresponsive pneumonia, especially in studies of immunocompromised patients, where the accuracy of mNGS detection can even reach 100% (Li et al., 2021). Therefore, early, sensitive and accurate diagnosis is essential to identify and confirm the presence of pathogens of pulmonary infections. Respiratory tract infections generally manifest as fever, chill, cough, sputum, and in severe cases, respiratory distress may occur. Imaging examinations such as high-resolution computed tomography (CT) may aid in the diagnosis (Tanaka et al., 2011), but the specific type of pathogenic infection is difficult to determine. Conventional PCR can only detect several common types of viruses, traditional pathogenic culture are time-consuming, easily contaminated and have a low positive rate. Fungal detection is more difficult. In most cases, it is difficult to obtain patient sputum for culture, making it difficult to diagnose the type of pathogenic infection. Bronchoalveolar lavage fluid (BALF) is a better source of specimens for detecting pathogenic species of pulmonary infections by obtaining alveolar surface cells, soluble material, microorganisms and parasites and may contain intact fungal cells through fiberoptic bronchoscopy (Springer et al., 2018). As a comprehensive and direct detection method, mNGS is difficult to become a first-line clinical detection method in a short period of time due to economic cost and other problems. It is still a very efficient pathogen screening method in the diagnosis of special patient populations such as immunodeficiency, and emerging infectious diseases (Chiu and Miller, 2019). It is the unbiased sequencing of all nucleic acids in a sample, and has unique advantages and attractions in the identification, typing, drug-resistant mutation detection and novel pathogen identification of pathogenic microorganisms.

Metagenomic next-generation sequencing (mNGS) has been applied to diagnose infection syndromes caused by multiple pathogens at multiple sites, including respiratory infections (Langelier et al., 2018; Shi et al., 2020; Bal et al., 2022), central nervous system infections (Zhang et al., 2021), and bloodstream infections (Goggin et al., 2020), due to its high sensitivity and specificity. And studies have also shown that mNGS is superior to traditional methods in detecting bacteria, fungi, mycoplasmas and viruses (Li et al., 2018; Fang et al., 2020; Huang et al., 2021). However, comparative studies using mNGS methods to detect pathogenic infection in BALF in haematological patients with respiratory symptoms undergoing allo-HSCT and chemotherapy are limited. Therefore, we conducted a retrospective study to compare and analyze mNGS detection of pathogenic species of infection in transplantation and chemotherapy patients to provide a reference for patient diagnosis and empirical treatment.

## 2 Methods

### 2.1 General information

The research protocol was approved by the Ethics Committee of the Affiliated Cancer Hospital of Zhengzhou University and the Third People’s Hospital of Zhengzhou. All patients provided informed consent for their inclusion in this study. For children, their parents and guardians provided informed consent. And the study was conducted in accordance with the Declaration of Helsinki. Hematologic patients with significant respiratory symptoms, poor empirical anti-infective treatment and difficult to obtain sputum samples who received mNGS of BALF were included in the analysis, and other specimens was excluded. We retrospectively analyzed the clinical characteristics of 84 patients with respiratory symptoms who received allo-HSCT and/or chemotherapy at the Affiliated Cancer Hospital of Zhengzhou University and the Third People’s Hospital of Zhengzhou from April 2020 to April 2022. The clinical characteristics were investigated and recorded. After the related contraindications excluded, bronchoscopy is performed by a specialized skilled physician, and the bronchoalveolar lavage fluid (BALF) is collected aseptically and processed promptly for testing. The mNGS method was used to detect all pathogens in the BALF. Routine Glucan (G) experiments, Galactomannan (GM) experiments, bacterial cultures were performed, G experiment is mainly for the detection of fungal cell wall component (1,3)-β-D-glucan, which is suitable for the early diagnosis of all deep fungal infections except *Cryptococcus* and *tuberculosis*, but cannot determine the species of fungus(There is no fungal infection below 70 pg/ml, 70-95 pg/ml is the observation period, continuous testing is given, and deep fungal infection is suspected if it is greater than 95pg/ml); GM experiment is for the detection of galactomannan, which is mainly suitable for the early diagnosis of invasive *Aspergillus* infections(Less than 0.65 ug/L is negative, greater than 0.85 ug/L is positive). And quantitative Polymerase Chain Reaction (qPCR) was used to detect the *cytomegalovirus (CMV, HHV-5)* and *Epstein-Barr virus (EBV, HHV-4)*. At the same time, imaging tests such as chest computed tomography (CT) and other means were used to assist the diagnosis.

### 2.2 Methods and process of mNGS

#### 2.2.1 Sample processing and sequencing

BALF samples were collected based on the standard clinical procedure. A total of 2 mL bronchoalveolar lavage fluid (BALF) was immediately inactived at 65°C for 30 minutes after collection. Next, 0.8mL BALF was placed in a 1.5-mL microcentrifuge tube with 2 g 0.5-mm glass beads. Tubes were agitated vigorously at 3000 rpm for 15 minutes on a vortex mixer attached to a horizontal platform. All samples were then centrifuged at 12000 rpm for 1 minute, and 0.6 mL sample was separated into a new 2.0-mL microcentrifuge tube followed by DNA or RNA extraction. TIANamp Micro DNA Kit (DP316, TIANGEN Biotech, Beijing, China) and QIAamp Viral RNA Mini Kit RNA extraction kit (QIAGEN, Hilden, Germany) were used for DNA or RNA extraction according to the manufacturers’ recommendation, respectively.

DNA libraries were constructed through DNA fragmentation, end-repair, adaptor ligation, and PCR amplification using One Shot Max DNA Library Prep Kit (PDM601, Nanjing Practice Medicine Dignostics Co., Ltd, Nanjing, China) and NGS library construction kit (2012B, Genskey, Tianjin) according to the protocol. For RNA libraries construction, 10-1000 ng of RNA was mixed with ribosomal RNA (rRNA) deletion probes for rRNA deletion. Fragmentation, the synthesis of the first and second strands, end-repair, adaptor ligation were performed using an RNA library construction kit (PDR803, Nanjing Practice Medicine Dignostics Co., Ltd, Nanjing, China). The constructed library was qualified by Agilent 2100 Bioanalyzeer system (Agilent Technologies, Santa Clara, CA, USA) and Qubit 4.0 (Thermo Fisher Scientific Inc, USA). The qualified double-strand DNA library was transformed into a single-stranded circular DNA library through DNA-denaturation and circularization. DNA nanoballs (DNBs) were generated from single-stranded circular DNA by rolling circle amplification (RCA). The DNBs were qualified using Qubit 4.0. Qualified DNBs were loaded into the flow cell and sequenced (50 bp, single-end) on the MGISEQ-200 platform.

#### 2.2.2 Criteria for the mNGS results and bioinformatic analysis

For all pathogens originally detected, the obvious sequence alignment abnormalities (for the detected species, genome coverage<1% and depth>2) and background polluted bacteria were first filtered out (Jain et al., 2015; Shi et al., 2020). We used these two metrics primarily to filter out false-positive bacteria resulting from data analysis, as species with low genomic coverage but higher depth are largely due to reads multi-alignment. High-quality sequencing data were generated by removing low-quality and short (length < 35 bp) reads using fastq software (Chen et al., 2018), followed by computational subtraction of human host sequences mapped to the human reference genome (hg38) using STAR alignment (Dobin et al., 2013). After the removal of low complexity and duplicated reads using PRINSEQ algorithms (Schmieder and Edwards, 2011), the remained data were classified by simultaneously aligning to in-house microbial genome databases, consisting of viruses, bacteria, fungi, and parasites, which were mainly downloaded from NCBI (ftp://ftp.ncbi.nlm.nih.gov/genomes/) using Kraken2 software (Wood et al., 2019). The sequencing data list was analyzed in terms of species-specific read number (SSRN), reads per million (RPM) and genome coverage (%).

In our study, we set negative control samples in each batch of sequencing. Pathogen screening is performed based on this NC. The nucleic acids of negative control were derived from pure HL60 cell lines. We use a background database to remove background noise in analysis. In addition, we set a negative control sample (NC) in each batch of sequencing. Pathogen screening criteria are as follows:

1. Intracellular Bacteria, such as Mycobacterium tuberculosis complex, Brucella, Legionella pneumophila and Nocardia, the results were considered positive if a species had a species-specific read number ≥1. Because the intracellular bacteria not a colonize microbial, and low possibility for contamination. At the same time, the nucleic acids of intracellular bacteria are difficult to extract.
2. For extracellular bacteria, viruses and parasites, a microorganism was always excluded as a routine if it was detected in BALF samples as the same as that in the negative control. However, the results of BALF samples were considered to be positive if the RPM (reads per million reads) for a microorganism were ≥ tenfold than that in the NC.
3. For fungi, the results of BALF samples were considered to be positive if the RPM (reads per million reads) for fungi were ≥ fivefold than that in the NC.

#### 2.2.3 Statistical analyses

Categorical variables were expressed as percentages. Comparative analysis of count data was conducted by Pearson’s test or Fisher’s exact test. Data analysis was performed with IBM SPSS Statistics 25.0 and Graphpad Prism 8.0.1 P < 0.05 was considered statistically significant.

## 3 Results

### 3.1 Characteristics of the patients

**Figure 1:**
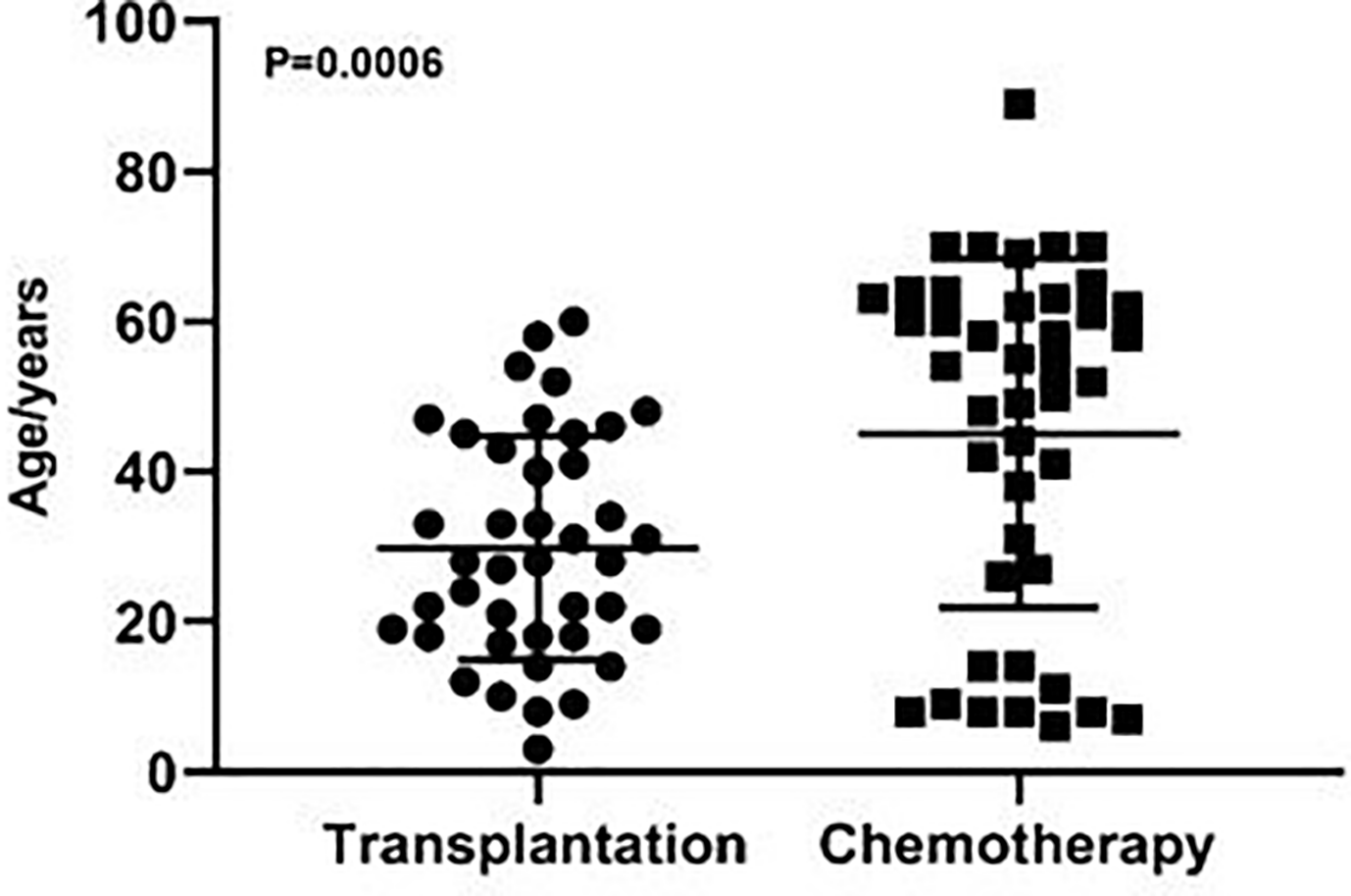
The age distribution of patients in the transplantation and chemotherapy groups. The patients in the transplant group were significantly younger than those in the chemotherapy group (P=0.0006).

Among the 84 patients, 41 patients received allo-HSCT and 43 patients received chemotherapy. Male and female patients were 22 and 19, 27 and 16, in the transplantation and chemotherapy groups, respectively. The transplantation group included 26 cases of acute myeloid leukemia (AML), 10 cases of acute lymphoblastic leukemia (ALL), 2 cases of aplastic anemia (AA), 3 cases of chronic myelomonocytic leukemia (CMML), and the chemotherapy group included 14 cases of AML, 7 cases of ALL, 10 cases of non-Hodgkin lymphomas (NHL), 9 cases of multiple myeloma (MM), 2 cases of AA, 1 case of chronic lymphocytic leukemia (CLL) (**Table 1**). The median age was 28 (3-60) and 54 (6-89) years in the transplantation and chemotherapy groups, respectively. The patients in the transplant group were significantly younger than those in the chemotherapy group, and the difference was statistically significant (P=0.0006) (**Figure 1**).

**Table 1:** Characteristics and prognosis of patients (N=84).

	Transplantation(N = 41)	Chemotherapy(N = 43)
Gender (n, %)		
Male	22 (53.7)	27 (62.8)
Female	19 (46.3)	16 (37.2)
Disease (n, %)		
AML	26 (63.4)	14 (32.6)
ALL	10 (24.4)	7 (16.3)
NHL	0	10 (23.3)
MM	0	9 (20.9)
AA	2 (4.9)	2 (4.7)
CMML	3 (7.3)	0
CLL	0	1 (2.2)
Conventional culture (n, %)		
Positive	8 (19.5)	6 (14.0)
Negative	33 (80.5)	37 (86.0)
mNGS (n, %)		
Negative	1 (2.1)	4 (8.9)
Single pathogen	8 (16.7)	12 (26.7)
Mixed pathogens	39 (81.3)	29 (64.4)
Mixed pathogen types (n, %)		
Bacteria - Virus	14 (35.9)	13 (44.8)
Fungi - Virus	6 (15.4)	5 (17.3)
Virus - Virus	13 (33.3)	2 (6.9)
Bacteria - Fungi - Virus	4 (10.2)	6 (20.8)
Bacteria - Fungi	1 (2.6)	1 (3.4)
Virus - Mycoplasma	1 (2.6)	0
Bacteria - Mycoplasma	0	1 (3.4)
Virus - Ureaplasma	0	1 (3.4)
Prognosis (n, %)		
Survive	28 (68.3)	31 (72.1)
Death	13 (31.7)	12 (27.9)
Died of infection		
Yes	10 (76.9)	2 (16.7)
No	3 (23.1)	10 (83.3)
Middle Age	28 (3-60)	54 (6-89)

AML, acute myeloid leukemia; ALL, acute lymphoblastic leukemia; NHL, non-Hodgkin lymphomas; MM, multiple myeloma; AA, aplastic anemia; CMML, chronic myelomonocytic leukemia; CLL, chronic lymphocytic leukemia; mNGS, metagenomic next generation sequencing.

### 3.2 Results of BALF detection in the transplantation and chemotherapy group

In the transplantation and chemotherapy groups, 41 and 43 tests were performed by conventional methods, respectively, and 48 (3 tests in 2 patients and 2 tests in 3 patients) and 45 (2 tests in 1 patient) tests were performed by mNGS, respectively. In the transplantation group, 8 cases (19.5%) and 47 cases (97.9%) were positive for BALF by conventional method detection and mNGS detection, respectively, and 6 cases (14.0%) and 41 cases (91.1%) in chemotherapy group, respectively. The detection rate of mNGS in both groups was significantly higher than that of conventional detection methods (all P<0.05). About the analysis of mNGS detection results, the genome coverage is the percentage of target bases that have been sequenced for a one time. Depth refers to the average sequencing depth of the coverage area can be defined theoretically as LN/G, where L is the read length, N is the number of reads and G is the coverage area length. We used these two metrics primarily to filter out false-positive bacteria resulting from data analysis. Additionally, in our past analysis, a database of colonizing microorganisms in different parts of healthy people was established, including common colonizing microorganisms and relative abundance ranges. If a microorganism is a colonized microorganism, we will consider its relative abundance in patient samples. If the relative abundance is higher than negative control and beyond the normal range in database, we consider that it may be a pathogen. Of course, we also need to consider the patient’s clinical features and medication situations.

**Figure 2:**
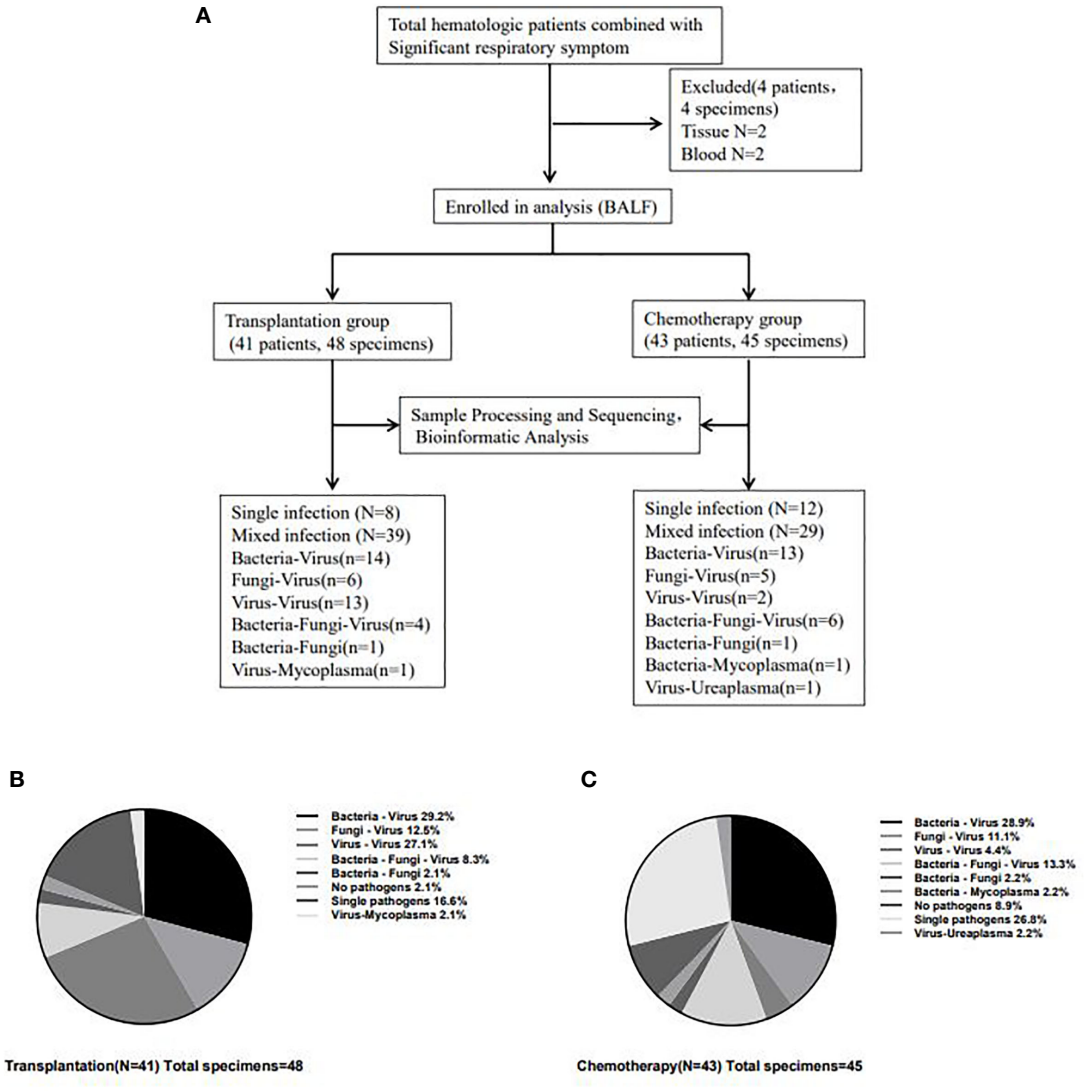
Detection of infectious pathogens by mNGS in transplantation group and chemotherapy group. The flow chart for inclusion of analyzed patients **(A)** and the most common type of infection in patients in the transplantation **(B)** and chemotherapy **(C)** groups was the mixed infection of bacteria-virus. And the mixed infection of virus-virus in the transplantation group was significantly higher than that in the chemotherapy group (27.1% *vs* 4.4%, P=0.003).

Among the patients with positive mNGS test in the transplantation group, there were 8 (16.7%) single pathogen infections, 39 (81.3%) mixed pathogen infections, including 14 (35.9%) mixed bacterial-viral infections, 6 (15.4%) mixed fungal-viral infections, 13 (33.3%) mixed viral-viral infections, 1 (2.6%) mixed bacterial-fungal infection, 1 (2.6%) mixed viral-mycoplasma infection, and 4 (10.2%) mixed bacterial-fungal-viral infections. Among the patients with positive mNGS test in the chemotherapy group, there were 12 (26.7%) single pathogen infections, 29 (64.4%) mixed pathogen infections, including 13 (44.8%) mixed bacterial-viral infections, 5 (17.3%) mixed fungal-viral infections, 2 (6.9%) mixed viral-viral infections, 1 (3.4%) mixed bacterial-fungal infection, 1(3.4%) mixed viral-ureaplasma infection, 1 (3.4%) mixed bacterial-mycoplasma infection and 6 (20.8%) mixed bacterial-fungal-viral infections. (**Table 1** and **Figures 2A-C**).

The most common type of infection in patients in the transplantation and chemotherapy groups was the mixed infection of bacteria-virus. Most patients in the transplantation group had mixed infections based on multiple viruses, with 42 cases of viral infections in the transplantation group and 30 cases of viral infections in the chemotherapy group, which were significantly higher in the transplantation group than in the chemotherapy group (χ^2 =^ 5.766, P=0.016). and the mixed infection of virus-virus in the transplantation group was significantly higher than that in the chemotherapy group (27.1% *vs* 4.4%, P=0.003), but the bacterial infection (χ^2 =^ 0.543, P=0.461) and fungal infection (χ^2 =^ 2.438, P=0.118) had no significant difference between the two groups. In addition, the proportion of single pathogen infection in the chemotherapy group was higher than that in the transplantation group, but there was no statistical difference (26.8% *vs* 16.6%, P=0.241).

**Figure 3:**
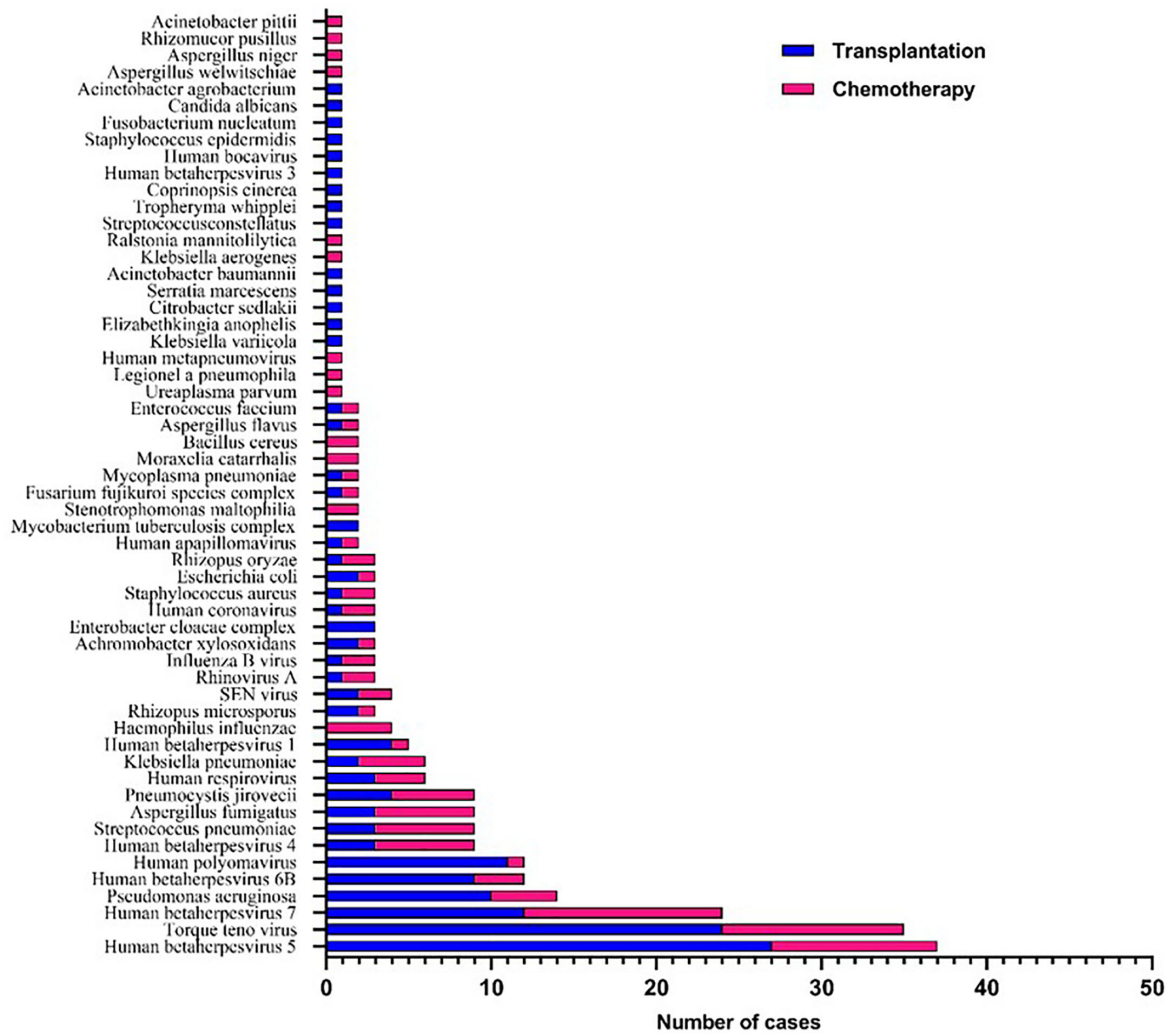
56 major pathogen species were detected in the transplantation and chemotherapy groups. *Human betaherpesvirus 5 (HHV-5)*, *torque teno virus* and *HHV-7* are the most common species of infection in the transplantation and chemotherapy groups.

**Figure 4:**
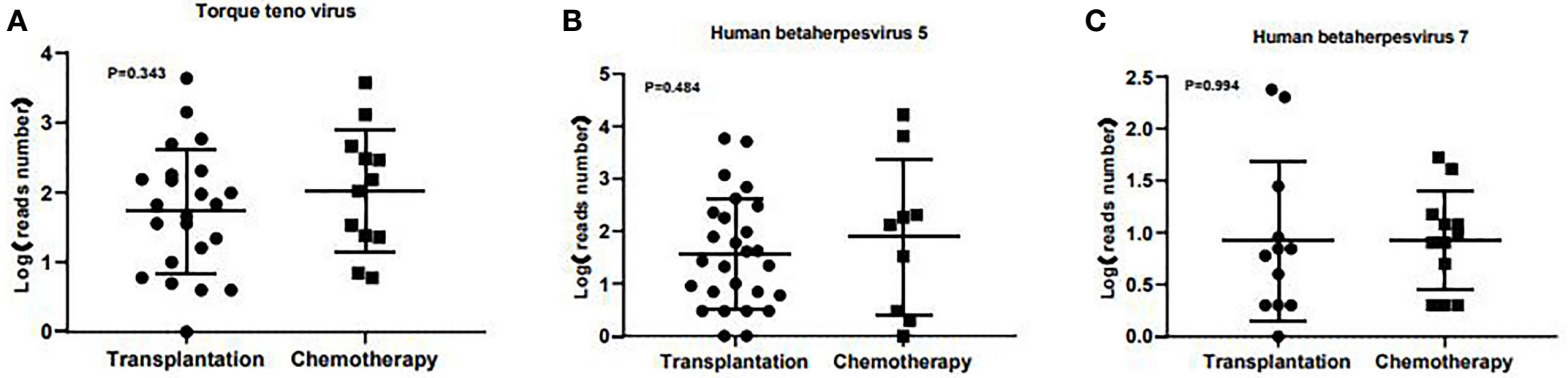
The number of reads detected by mNGS for *torque teno virus*, *HHV-5* and *HHV-7* in the transplantation and chemotherapy groups. There was no significant difference in the number of reads detected by mNGS for *torque teno virus* **(A)**, *HHV-5* **(B)** and *HHV-7* **(C)** among the common infectious pathogens in the two groups.

A total of 56 major pathogen species were detected in both groups, of which *human betaherpesvirus 5 (HHV-5), torque teno virus, human betaherpesvirus 7 (HHV-7), human polyomavirus, pseudomonas aeruginosa* and *human betaherpesvirus 6B (HHV-6B)* were the six most common pathogen species in transplantation group, and *HHV-7*, *torque teno virus*, *HHV-5*, *streptococcus pneumoniae, aspergillus fumigatus* and *HHV-4* were the six most common pathogen species in chemotherapy group (**Figure 3**). There was no significant difference in the number of reads detected by mNGS for *HHV-5, torque teno virus and HHV-7* among the common infectious pathogens in the two groups (**Figure 4**). *Pseudomonas aeruginosa and Streptococcus pneumoniae* were the most common bacterial infections in the transplantation and chemotherapy groups, respectively. *Aspergillus* was the most common fungal infection in both groups. (Supplementary table)

### 3.3 Results of BALF Detection in the early and late stages of transplantation

**Figure 5:**
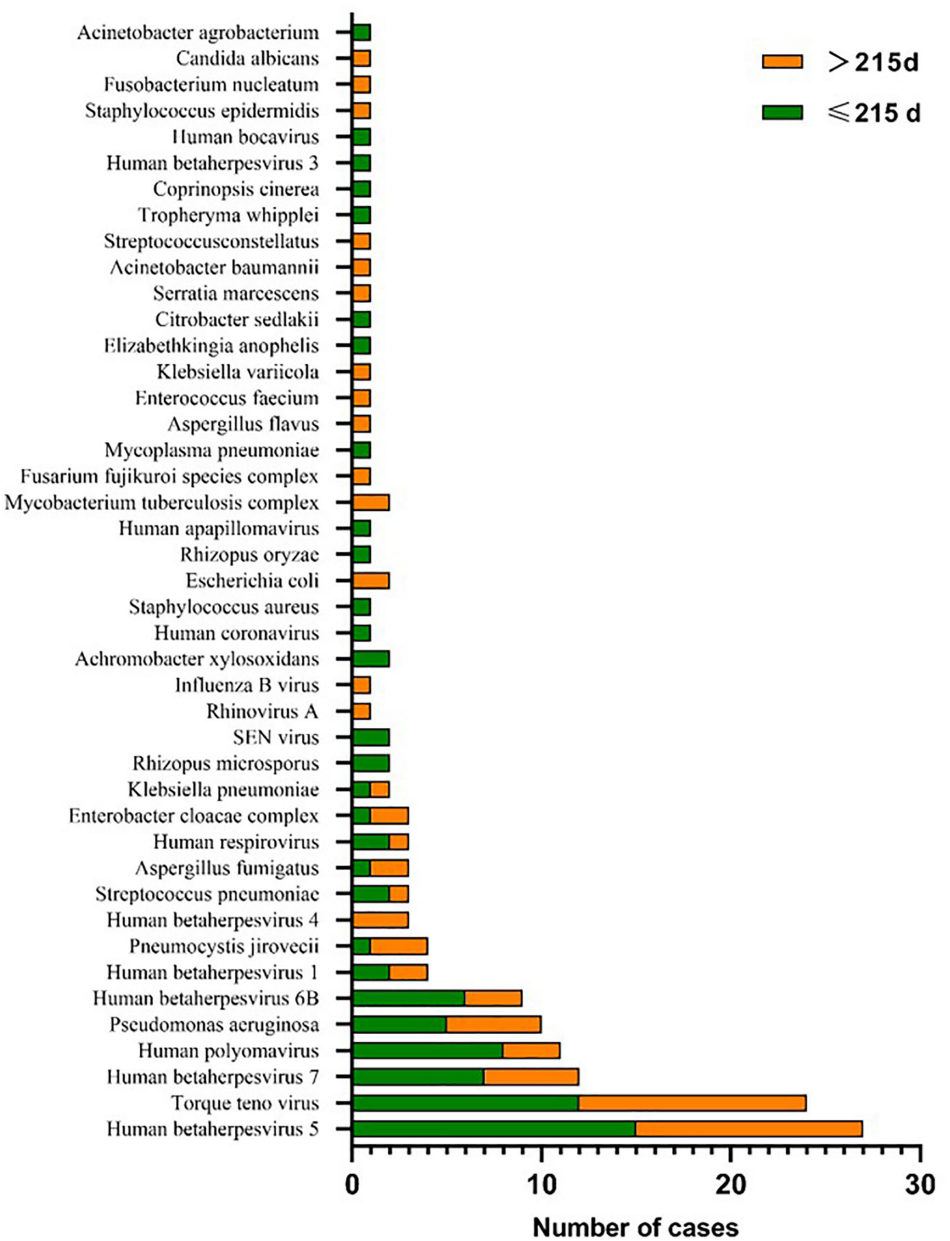
The major pathogen species were detected in the early and late stages of transplantation. *HHV-5*, *torque teno virus* and *HHV-7* are the most common species of infection in the early and late stages of transplantation.

**Figure 6:**
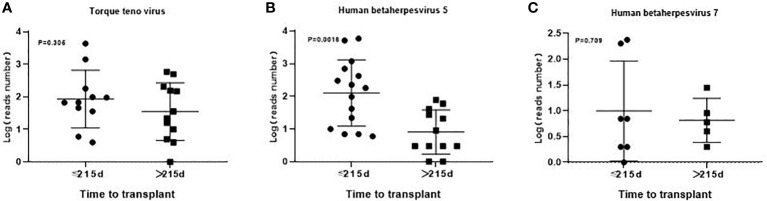
The number of reads detected by mNGS for *torque teno virus*, *HHV-5* and *HHV-7* in the early and late stages of transplantation. There was no significant difference in the number of reads detected by mNGS for *torque teno virus* **(A)**, *HHV-7* **(C)** except *HHV-5* **(B)** and among the common infectious pathogens between the early and late stages of transplantation.

In the transplantation group, the median time between BALF detection and transplantation was 215 (20-1853) days. The detection results within 215 days showed 21 cases of virus infection, 11 cases of bacteria and 5 cases of fungi. There were 20 cases of virus infection,13 cases of bacteria and 6 cases of fungi in the detection results greater than 215 days. There was no obvious difference in the types of pathogens infected in the early and late stages of transplantation. Additionally, there was no significant difference in the number of reads detected by mNGS for *torque teno virus* and *HHV-7* except *HHV-5* among the common pathogens between the early and late stages of transplantation (**Figures 5**, **6**).

### 3.4 Treatment and prognosis

Corresponding anti-infection and symptomatic and supportive treatment are given according to the patient’s pathogen test results, drug susceptibility and drug resistance type. Among 40 infected patients in the transplantation group, 30 had improved respiratory symptoms after treatment and 10 had no significant improvement, and among 39 patients in the chemotherapy group, 37 had significant improvement in symptoms after treatment and 2 had no significant improvement. As of April 2022, a total of 13 patients died in the transplantation group,3 patients died due to organ failure caused by GVHD, 10 patients died mainly from severe pneumonia and respiratory failure. 12 patients died in the chemotherapy group, 2 cases died of severe pneumonia, 10 patients died mainly due to advanced age combined with progression of the primary disease. The proportion of death due to pulmonary infection was significantly higher in the transplantation group than in the chemotherapy group (76.9% *vs* 16.7%, χ^2 =^ 9.077, P=0.003) (**Table 1**).

## 4 Discussion

In this study, we compared bronchoalveolar lavage fluid mNGS sequencing results in hematologic patients receiving allo-HSCT and chemotherapy combined with respiratory symptoms. In general, the positive detection rate in the transplantation and chemotherapy group were 97.9% (47/48) and 91.1% (41/45), respectively. And the mNGS also outperforms traditional methods in pathogen species detection. mNGS can detect a variety range of uncommon viruses, bacteria and fungi, such as *human bocavirus, ralstonia mannitolilytica, tropheryma whipplei, aspergillus welwitschiae.*

Because mNGS is culture-independent, ex vivo pathogen viability has no effect on assay results, making it easier to assess slow-growing, difficult-to-cultivate pathogens, and pathogens that are lethal due to antibiotic exposure. This is also the advantage of mNGS over whole genome sequencing (WGS) (Wang et al., 2020; Diao et al., 2022). Despite the appeal of culture-independent mNGS, many issues remain to be addressed. In particular, how to distinguish pathogenic microorganisms from colonizing microorganisms when mNGS is used for the diagnosis of pneumonia. This requires us to pay strict attention to the handling of specimens, and minimize the impact of contamination by removing human nucleic acidsand avoiding external sources of contamination during the operation (Marotz et al., 2018; Charalampous et al., 2019; Weyrich et al., 2019). In addition, many bioinformatics methods are also available for contamination removal. The most common decontamination methods for bioinformatics involve filtering sequences below a relative abundance threshold, but there is a risk of true low-frequency sequences being discarded and a large number of contaminating sequences interfering with downstream analyses (Weyrich et al., 2019). Another simple method is to subtract the number of sequences that appear in the negative control (Wilson et al., 2018). In general, quantitative or semi-quantitative statistical analysis is used to differentiate between true pathogens and colonizers in respiratory mNGS assays. mNGS detection of pulmonary infectious pathogens typically employs metrics such as relative abundance at the genus level, normalized to sequences per million, sequences aligned to target microorganisms, and genome coverage in non-overlapping regions (Larsson et al., 2018; Li et al., 2018; Huang et al., 2020). Langelier et al. distinguish pathogens and background microorganisms by z-score in bioinformatics analysis (Langelier et al., 2018). Additionally, a study developed a rule-based model and a logistic regression model to distinguish pathogenic and colonizing microorganisms, both models achieved accuracies of 95.5% (Langelier et al., 2018).Although some of them we cannot confirm whether they are colonizing or pathogenic bacteria, they still need to be treated promptly in conjunction with clinical symptoms, immune function and underlying diseases. Early and specific diagnosis of pathogens and early initiation of specific antifungal therapy are directly associated with improved clinical outcomes (Drgona et al., 2014).

Mixed pathogen infection is a common type of infection in immunocompromised patients (Di Pasquale et al., 2019; Wang et al., 2019). Conventional detection methods are difficult to detect multiple pathogens simultaneously (Jain et al., 2015). As a highly sensitive analytical method, mNGS can overcome the limitations of traditional detection methods and provide more valuable reference results for the detection of various pathogens (Chiu and Miller, 2019; Gu et al., 2019; Wang et al., 2019). In this study, the results of mNGS detection in both groups of patients showed that virus-based mixed infections were the most common. The detection rate of viruses and mixed virus-virus infections were significantly higher in the transplantation group than in the chemotherapy group, which may be related to the more pronounced immunosuppression and longer duration of immunodeficiency in transplantation patients (Sahin et al., 2016; Green, 2017). There was no significant difference in the proportion of bacterial and fungal infections between the two groups. There were also no significant differences in the types of pathogenic bacteria that infected patients during the different transplantation periods. This suggests that we should not only take preventive measures against viral infection as soon as possible for transplant patients, but also empirically give combined anti-infective therapy when patients have respiratory symptoms to reduce adverse outcomes caused by pulmonary infection (Bondeelle and Bergeron, 2019).

In terms of empirical anti-infection treatment of patients, *HHV5 (CMV)* was the most common virus infection in the transplantation group, while *HHV7* was the most common in the chemotherapy group. Among the bacterial infections, *Pseudomonas aeruginosa* and *Streptococcus pneumoniae* were most common in the transplantation and chemotherapy groups, respectively, which may be related to the pretreatment regimen medication and long application of immunosuppressive agents in patients in the transplantation group, and it is prone to the infection of Gram-negative bacilli (Zhou and Moore, 2021). In contrast, the chemotherapy group was dominated by common Gram-positive cocci. Similar findings have also shown that *pseudomonas aeruginosa* is more prevalent after allo-HSCT and in immunocompromised patients, but is not an independent risk factor for it (Di Pasquale et al., 2019; Zhou and Moore, 2021). Among the fungal infections, *Aspergillus* infections were more common in both groups, and *Mucor* and *Candida* were less common. Previous studies have also shown that *Aspergillus* are the most common fungus responsible for pulmonary infections in human (Maschmeyer and Donnelly, 2016). This suggests that for patients with hematologic diseases, the presence of respiratory tract infections can be used as a basis for empirical anti-infective therapy when the pathogenic organism is not identified. Firstly, for transplantation patients, the use of immunosuppressive drugs should be reduced under the control of graft-versus-host disease (GVHD). Secondly, patients with viral infection should be given antiviral combined immunoglobulin therapy in a timely manner. Finally, stem cell support therapy, such as donor lymphocyte infusion (DLI) (Kishi et al., 2000), *Epstein-Barr virus* specific cytotoxic T lymphocyte (EBV-CTL) (Kuzushima et al., 1996), *cytomegalovirus (CMV)*-specific cytotoxic T lymphocyte (CMV-CTL) (Wang et al., 2022) are given when conditions permit. In addition, measures to prevent viral infection, such as the application of rituximab, can also be given in the early stage of transplantation (Stocker et al., 2020; Walti et al., 2021). Although *HHV7* is the more common viral infection in the chemotherapy group, and primary *HHV7* infections are mostly associated with acute rash and roseola in children, *HHV-7* is 75% homologous to *CMV*, and immunosuppressive therapy in chemotherapy patients creates favorable conditions for reactivation of *HHV-7*, which may increase the risk of *CMV* infectious disease, an opportunistic infectious agent, and *CMV* reactivation is a life-threatening infectious complication that is associated with an increased risk of overall patient death (Osman et al., 1996; Hill et al., 2019; Einsele et al., 2020). Therefore, it is also important to give appropriate antiviral treatment in a timely manner. For the treatment of bacterial infections, two flora-sensitive antibiotics were selected for treatment, and no relevant contraindications were given to multiple antibiotic combinations when necessary to avoid drug-resistant bacteria. As for the treatment of fungal infections, since *Aspergillus* infections are the most common and immunocompromised patients are an important cause of morbidity and mortality of invasive fungal diseases, it is more important to treat them actively and early (Robenshtok et al., 2007; Drgona et al., 2014), and anti-infective treatment should be based on liposomal amphotericin B (LAMB) and voriconazole and other azole drugs (Herbrecht et al., 2002; Cornely et al., 2007). At the same time, reducing the dosage of hormones is also a crucial condition for improving the prognosis of invasive aspergillosis (Walsh et al., 2008).

By the end of follow-up, the patients who died in the transplantation group were mainly suffering from pulmonary infections, which may be related to the fact that the patients were on higher doses of immunosuppressants and the anti-infective treatment was not effective. In contrast, patients in the chemotherapy group had relatively low dosages of immunosuppressants and better anti-infective treatment, and the patients who died were mainly due to advanced age and relapse of the primary disease. In this regard, we should take different treatment measures for patients with chemotherapy and transplantation co-infection. Transplantation patients should focus on enhancing the patient’s immunity while anti-infection treatment, while chemotherapy patients should focus more on the treatment of the primary disease while the infection is under control. Of course, since this study is a single-center retrospective study, it does not yet have the exact value and significance of guiding treatment, and a large-scale multicenter prospective study is needed for more detailed treatment protocols to determine the guiding value of mNGS detection of bronchoalveolar lavage fluid in the treatment of patients with hematologic diseases.

In conclusion, although mNGS is highly sensitive and indiscriminate in the detection of pathogenic bacteria, it can also produce false positive results. Therefore, it is necessary for clinicians to cooperate closely with the testers to combine the test results with clinical symptoms to make a more accurate assessment of the patient’s condition and to take comprehensive, rational and effective therapeutic measures. While taking into account the timely and accurate treatment of the primary disease and avoiding the excessive application of antibiotics, reducing the death caused by severe pneumonia, and making full use of the advantages of mNGS to provide good guidance for the application of clinical antibiotics, thus improving the prognosis of patients with hematological disease combined with respiratory symptoms.

## Data availability statement

The original contributions presented in the study are included in the article/**Supplementary Material**. Further inquiries can be directed to the corresponding authors.

## Ethics statement

This study was reviewed and approved by the Affiliated Cancer Hospital of Zhengzhou University and the Third People’s Hospital of Zhengzhou. Written informed consent to participate in this study was provided by the participants’ legal guardian/next of kin.

## Author contributions

BZ, JZ and YS designed the study. RG, QW, XJ and ZL implemented this research. BZ, JW, LH, LZ and XL collected medical records. BZ, YS, XL and JZ drafted the manuscript. All the authors participated in the revision of the manuscript. All authors contributed to the article and approved the submitted version.

## Acknowledgments

We would like to thank all the patients for their cooperation and the Department of Hematology, the Affiliated Cancer Hospital of Zhengzhou University and The Third People’s Hospital of Zhengzhou. Thanks to Nanjing Practice Medicine Diagnostics. Co., Ltd and Beijing Genskey Co., Ltd, China for providing sequencing technical support.

## Conflict of interest

The authors declare that the research was conducted in the absence of any commercial or financial relationships that could be construed as a potential conflict of interest.

The handling editor [LA] declared a shared parent affiliation with the author(s) [BZ, RG, QW, XJ, ZL, JW, LH, LZ, XL, JZ] at the time of review.

## Publisher’s note

All claims expressed in this article are solely those of the authors and do not necessarily represent those of their affiliated organizations, or those of the publisher, the editors and the reviewers. Any product that may be evaluated in this article, or claim that may be made by its manufacturer, is not guaranteed or endorsed by the publisher.

## Abbreviations

BALF, central nervous system; mNGS, metagenomic next-generation sequencing; HHV-5, human betaherpesvirus 5; allo-HSCT, allogeneic hematopoietic stem cell transplantation; CT, computed tomography; EBV, Epstein-Barr virus; AML, acute myeloid leukemia; ALL, acute lymphoblastic leukemia; AA, aplastic anemia; CMML, chronic myelomonocytic leukemia; NHL, non-Hodgkin lymphomas; MM, multiple myeloma; CLL, chronic lymphocytic leukemia; GVHD, graft versus host disease; DLI, donor lymphocyte infusion; CMV-CTL, cytomegalovirus-specific cytotoxic T lymphocyte; EBV-CTL, Epstein-Barr virus specific cytotoxic T lymphocyte; LAMB, liposomal amphotericin B.
