# Supplementary material for: Comparing the application of mNGS after combined pneumonia in hematologic patients receiving hematopoietic stem cell transplantation and chemotherapy: A retrospective analysis
Source: Front Cell Infect Microbiol. 2022 Sep 21;12:969126. doi: 10.3389/fcimb.2022.969126 (PMC9532739; doi:10.3389/fcimb.2022.969126)
Supplement: Supplementary file 1 [file Table_1.docx]

Supplementary Table1

Pathogen species detected by mNGS in patients in the transplantation and chemotherapy groups.

|  | Transplantation（n) | chemotherapy（n) |
| --- | --- | --- |
| Human betaherpesvirus 5 | 27 | 10 |
| Torque teno virus | 24 | 11 |
| Human betaherpesvirus 7 | 12 | 12 |
| Pseudomonas aeruginosa | 10 | 4 |
| Human betaherpesvirus 6B | 9 | 3 |
| Human polyomavirus | 11 | 1 |
| Human betaherpesvirus 4 | 3 | 6 |
| Streptococcus pneumoniae | 3 | 6 |
| Aspergillus fumigatus | 3 | 6 |
| Pneumocystis jirovecii | 4 | 5 |
| Human respirovirus | 3 | 3 |
| Klebsiella pneumoniae | 2 | 4 |
| Human betaherpesvirus 1 | 4 | 1 |
| Haemophilus influenzae | 0 | 4 |
| Rhizopus microsporus | 2 | 1 |
| SEN virus | 2 | 2 |
| Rhinovirus A | 1 | 2 |
| Influenza B virus | 1 | 2 |
| Achromobacter xylosoxidans | 2 | 1 |
| Enterobacter cloacae complex | 3 | 0 |
| Human coronavirus | 1 | 2 |
| Staphylococcus aureus | 1 | 2 |
| Escherichia coli | 2 | 1 |
| Rhizopus oryzae | 1 | 2 |
| Human apapillomavirus | 1 | 1 |
| Mycobacterium tuberculosis complex | 2 | 0 |
| Stenotrophomonas maltophilia | 0 | 2 |
| Fusarium fujikuroi species complex | 1 | 1 |
| Mycoplasma pneumoniae | 1 | 1 |
| Moraxelia catarrhalis | 0 | 2 |
| Bacillus cereus | 0 | 2 |
| Aspergillus flavus | 1 | 1 |
| Enterococcus faecium | 1 | 1 |
| Ureaplasma parvum | 0 | 1 |
| Legionel a pneumophila | 0 | 1 |
| Human metapneumovirus | 0 | 1 |
| Klebsiella variicola | 1 | 0 |
| Elizabethkingia anophelis | 1 | 0 |
| Citrobacter sedlakii | 1 | 0 |
| Serratia marcescens | 1 | 0 |
| Acinetobacter baumannii | 1 | 0 |
| Klebsiella aerogenes | 0 | 1 |
| Ralstonia mannitolilytica | 0 | 1 |
| Streptococcusconstellatus | 1 | 0 |
| Tropheryma whipplei | 1 | 0 |
| Coprinopsis cinerea | 1 | 0 |
| Human betaherpesvirus 3 | 1 | 0 |
| Human bocavirus | 1 | 0 |
| Staphylococcus epidermidis | 1 | 0 |
| Fusobacterium nucleatum | 1 | 0 |
| Candida albicans | 1 | 0 |
| Acinetobacter agrobacterium | 1 | 0 |
| Aspergillus welwitschiae | 0 | 1 |
| Aspergillus niger | 0 | 1 |
| Rhizomucor pusillus | 0 | 1 |
| Acinetobacter pittii | 0 | 1 |

Supplementary table2

mNGS results of per patient in the transplantation group

| Sample ID | Microorganisms and reads number |
| --- | --- |
| MBX10838 | DNA：Human polyomavirus 9；HHV-5 1 |
| MBX18794 | DNA：Streptococcus pneumoniae 1275；Pseudomonas aeruginosa 13；Human polyomavirus 29；TTV 22；  HHV-7 4；HHV-5 3; Mycobacterium tuberculosis complex 2 |
| NGS293 | DNA：Aspergillus fumigatus 84； HHV-1 1 |
| NA* | DNA：TTV 154；HHV-4 29 |
| 20PM100944 | DNA：Human polyomavirus 707479；HHV-5 5886 |
| 20PM101285 | DNA：Human polyomavirus 1884414； TTV 46 ； HHV-5 10 ；HHV-7 7 |
| 20JS060676 | DNA：HHV-5 97； TTV 67； Human polyomavirus4 10；  RNA：Human coronavirus 4236；TTV 32；HHV-5 24; Human respirovirus 4；Human polyomavirus 1 |
| 20PM100954 | DNA：HHV-4 6 |
| 20PM100953 | DNA：Escherichia coli 211；TTV 150；HHV-5 21 |
| MBX22310 | DNA：Pseudomonas aeruginosa 6；TTV 494；HHV-5 9 |
| NGS179 | DNA：Pseudomonas aeruginosa 25975；Klebsiella variicola 3；HHV-5 78；TTV 1；  RNA：Pseudomonas aeruginosa 39034；Klebsiella variicola 5；Rhinovirus A 102；HHV-5 54 |
| 20PM101169 | DNA：Pseudomonas aeruginosa 53；HHV-5 700；HHV-7 238；HHV-6 62；TTV 36 |
| 20PM101119 | DNA：Staphylococcus epidermidis 249 |
| 20PM101204 | DNA：Pseudomonas aeruginosa 1768；Fusobacterium nucleatum 572；Pneumocystis jirovecii 51；Candida albicans 11 |
| 20PM101225 | DNA：Acinetobacter agrobacterium 6302；WU Human polyomavirus 3865；HHV-5 421 |
| 20JS057950 | DNA：Pseudomonas aeruginosa 17；Elizabethkingia anophelis 7；Achromobacter xylosoxidans 39；Aspergillus fumigatus 2；TTV 6 |
| 20JS057953 | DNA：Streptococcus pneumoniae 3；HHV-5 304；HHV-7 7 |
| NGS207 | DNA：TTV 10；HHV-5 3; |
| NGS150 | DNA：Pneumocystis jirovecii 26；Fusarium fujikuroi species complex 3；TTV 585； |
| NGS003 | DNA：Rhizopus microsporus 10；RNA：Rhizopus microsporus 24；Human respirovirus 6430 |
| NGS326 | DNA：Enterobacter cloacae complex 511；Rhizopus microsporus 2700982； Rhizopus oryzae 750547；Coprinopsis cinerea 12123；HHV-5 5112；TTV 156；HHV-7 201；HHV-6B 182；Human polyomavirus 10 |
| NGS398 | DNA：Pneumocystis jirovecii 223；Human polyomavirus 1726；HHV-5 22；SEN virus 16； HHV-6B 5 |
| NGS400 | DNA：Pseudomonas aeruginosa 53； Achromobacter xylosoxidans 28； Citrobacter sedlakii 15 |
| NGS438 | DNA：HHV-3 7236；HHV-1 56；HHV-5 7; HHV-6B 2 |
| NGS028 | DNA：Enterobacter cloacae complex 6161； Klebsiella pneumoniae 243； Serratia marcescens 24； HHV-6B 64 |
| NGS063 | DNA：TTV 36；HHV-5 41；HHV-7 28；HHV-4 9 |
| NGS081 | DNA：Staphylococcus aureus 2046；TTV 4350；Human polyomavirus 2174；HHV-5 180 |
| NGS108 | DNA：Pseudomonas aeruginosa 796519；Klebsiella pneumoniae 6；TTV 1416； |
| NGS080 | DNA：Pseudomonas aeruginosa 2591； |
| NGS076 | DNA：HHV-5 1184；SEN virus 1184 |
| 22PM200196 | DNA: Enterobacter cloacae complex 1421； |
| NGS248 | negative |
| NGS304 | DNA: TTV 99；Human apapillomavirus 45；HHV-5 42； HHV-7 1; HHV-6B 1； Mycoplasma pneumoniae 1； |
| NGS461 | DNA: Tropheryma whipplei 4；HHV-5 7；TTV 4；RNA: negative |
| NA* | DNA：HHV-1 2724；TTV 180；HHV-5 6；HHV-7 2；RNA：HHV-1 183；TTV 89：HHV-5 3；HHV-7 1 |
| NGS357 | DNA: TTV 205； HHV-5 60; HHV-6B 11; HHV-7 9 |
| NGS405 | DNA： Escherichia coli 2214；TTV 16 |
| NGS466 | DNA：Streptococcusconstellatus 17；TTV 5；Mycobacterium tuberculosis complex 25 |
| NGS062 | DNA：HHV-5 3 |
| NGS052 | DNA：HHV-7 2；HHV-6B 2；HHV-5 1；RNA：Human respirovirus 1816；Influenza B virus 5 |
| 22PM101029 | DNA：Aspergillus flavus 5；HHV-1 23； |
| 22PM101016 | DNA：Acinetobacter baumannii 2； |
| NGS119 | DNA：Streptococcus pneumoniae 41；TTV 95；HHV-7 2；HHV-6B 1 |
| NM21D0475 | DNA: Pneumocystis jirovecii 341；HHV-5 27；Human polyomavirus 3; |
| NM21D0856 | DNA: HHV-5 227 |
| NM21D0359 | DNA: Enterococcus faecium 6182；Pseudomonas aeruginosa 43；Aspergillus fumigatus 3；TTV 4；HHV-7 6；HHV-5 3 |
| NM21D0519 | DNA: Human polyomavirus 65508；TTV 68 |
| 22PM200104 | DNA: Human bocavirus 998 |

*Sequencing data was corrupted

Supplementary table3 mNGS results of per patient in the chemotherapy group

| Sample ID | Microorganisms and reads number |
| --- | --- |
| NGS042 | DNA：Bacillus cereus 5； Streptococcus pneumoniae 2；TTV 153；RNA：Bacillus cereus 14；TTV 36 |
| NGS043 | DNA：negative；RNA：negative |
| NGS135 | DNA：Streptococcus pneumoniae 56； RNA：Streptococcus pneumoniae 14 |
| NGS153 | DNA：Klebsiella aerogenes 1263； Human apapillomavirus 21；RNA： Human respirovirus 2679 |
| NGS154 | DNA：HHV-4 156； HHV-7 5 |
| NGS221 | DNA: Pseudomonas aeruginosa 22；Pneumocystis jirovecii 2371; RNA: Pneumocystis jirovecii 5109 |
| NGS189 | DNA：Moraxelia catarrhalis 14；Escherichia coli 5；Aspergillus fumigatus 1；HHV-7 12；HHV-6B 1 |
| NGS258 | DNA: negative ；RNA: Human respirovirus 819845 |
| NGS262 | DNA: Pseudomonas aeruginosa 33816；Klebsiella pneumoniae 831； HHV-4 41 |
| NGS289 | DNA：Pneumocystis jirovecii 10；HHV-5 3；HHV-7 2；RNA：Pneumocystis jirovecii 20；HHV-5 3；HHV-7 1 |
| 20PM101168 | negative |
| 20PM00388 | DNA：Pneumocystis jirovecii 16 |
| NGS334 | DNA：Pseudomonas aeruginosa 8405；TTV 306； HHV-7 10；RNA: Pseudomonas aeruginosa 1468 |
| NGS339 | DNA: Haemophilus influenzae 203374； TTV 1311；HHV-5 205；HHV-6B 33；HHV-7 15；  RNA：Haemophilus influenzae 727；Rhinovirus A 137； TTV 9；Human metapneumovirus 102；HHV-5 2 |
| NGS350 | DNA：Klebsiella pneumoniae 5391；Aspergillus welwitschiae 155；Aspergillus niger 140；Rhizopus oryzae 176； TTV 463； Human polyomavirus 4；HHV-7 2; RNA: Klebsiella pneumoniae 3；Rhizopus oryzae 11 |
| NGS354 | DNA: HHV-1 1263；TTV 24； HHV-7 53； HHV-6B 4; HHV-5 33; HHV-4 4;  RNA: HSV1 2441; Human coronavirus 1207； TTV 124； HHV-5 230; HHV-7 44; HHV-6B 3; HHV-4 35 |
| NGS368 | DNA: Aspergillus fumigatus 3 |
| NGS384 | DNA： Aspergillus fumigatus 1；TTV 3769；RNA： TTV 464；Rhinovirus A 283 |
| NGS044 | DNA：Streptococcus pneumoniae 13457；TTV 294；HHV-7 41 |
| NGS390 | negative |
| NGS393 | DNA：Staphylococcus aureus 16；Pneumocystis jirovecii 1707；TTV 23； HHV-4 2；  RNA：Pneumocystis jirovecii 1615；Human coronavirus 83；HHV-4 2 |
| NGS399 | DNA：Fusarium fujikuroi species complex 1077； RNA：Human respirovirus 2268 |
| NGS450 | DNA： Streptococcus pneumoniae 40；Pseudomonas aeruginosa 23；Pneumocystis jirovecii 10377；SEN virus 48 |
| NGS454 | DNA：Ralstonia mannitolilytica 104；RNA：Influenza B virus 10658 |
| NGS003 | DNA：Rhizomucor pusillus 8；RNA: Rhizomucor pusillus 54； Influenza B virus 20 |
| NGS006 | DNA：Rhizopus microsporus 1 |
| NGS010 | DNA：HHV-7 12；Ureaplasma parvum 21 |
| NGS023 | DNA：Aspergillus fumigatus 34 |
| NGS024 | DNA：Streptococcus pneumoniae 4；Mycoplasma pneumoniae 15486 |
| NGS041 | DNA：Stenotrophomonas maltophilia 392；Achromobacter xylosoxidans 15；Haemophilus influenzae 7；HHV-5 6495 |
| NGS042 | DNA：Klebsiella pneumoniae 3379； HHV-5 182 |
| NGS045 | DNA：HHV-5 16545；RNA：HHV-5 1903 |
| NGS056 | DNA：Legionel a pneumophila 4895；Aspergillus fumigatus 7；SEN virus 3 |
| NGS095 | DNA：Moraxelia catarrhalis 4；TTV 34；HHV-4 3；HHV-5 2 |
| NGS097 | DNA：Stenotrophomonas maltophilia 12842；Klebsiella pneumoniae 6351；HHV-7 2 |
| NGS144 | DNA: Haemophilus influenzae HHV-5 1 |
| NGS162 | DNA: Acinetobacter pittii 15；HHV-5 131；TTV 7；HHV-7 8 |
| NM21D0499 | DNA：Enterococcus faecium 144 |
| NM21D0647 | DNA：Streptococcus pneumoniae 21 |
| NM21D0946 | DNA：Aspergillus flavus 2；Rhizopus oryzae1；TTV 6；HHV-4 12 |
| NM21D1196 | DNA：TTV 105 |
| NM22D0290 | negative |
| NM21D0432 | DNA：Aspergillus fumigatus 2 |
| NM22D0061 | DNA：Haemophilus influenzae 39；Bacillus cereus 13；HHV-7 8 |
| NM22D0104 | DNA：Staphylococcus aureus 12865 |
